# P-1488. Attitudes and decision making about older adult vaccination – results from a literature review to inform the design of a qualitative research study

**DOI:** 10.1093/ofid/ofaf695.1672

**Published:** 2026-01-11

**Authors:** Gitte Lee Mortensen, Oliver Martyn, Mandira Kar

**Affiliations:** AnthroConcult, Aarhus, Midtjylland, Denmark; Sanofi Vaccines, Copenhagen, Hovedstaden, Denmark; Research Partnership, London, England, United Kingdom

## Abstract

**Background:**

Vaccination in older adults is crucial due to age-related immunosenescence and higher morbidity and mortality from infectious diseases. Despite the availability of effective vaccines for diseases such as influenza, pneumococcal disease, shingles, and respiratory syncytial virus (RSV), uptake remains suboptimal. This literature study explored the attitudes and decision-making processes related to older adult vaccination in Western countries, particularly post-COVID-19.

**Methods:**

A systematic literature search in PubMed focused on eliciting peer-reviewed articles published between 2020-2024, using terms related to vaccine hesitancy, acceptance, attitudes, and behaviors among older adults in Western countries. 28 relevant studies were included, providing insights into general attitudes, specific vaccine uptake, and the impact of the COVID-19 pandemic on vaccination behaviors. The findings informed the design of an ongoing study exploring this topic in depth using qualitative methods in the US and Europe.

**Results:**

The literature study identified several key drivers and barriers to vaccination. Drivers included awareness of vaccine-preventable diseases, perceptions of susceptibility, confidence in vaccines and healthcare professionals, convenience, preventive health behaviors, and healthcare provider recommendations. Barriers encompassed lack of awareness, complacency, confidence issues, structural constraints, and lack of healthcare provider recommendations. The COVID-19 pandemic influenced vaccination attitudes, increasing awareness and uptake for some vaccines while also contributing to vaccine fatigue and hesitancy.

**Conclusion:**

Understanding the factors influencing older adult vaccination is essential for developing targeted interventions to ensure informed decision making on older adult vaccination, in a future context of an increasing number of adult vaccinations. Strategies should focus on enhancing awareness, building trust in vaccines and healthcare providers, and addressing structural barriers. The findings underscore the need for continued research, particularly qualitative studies, to explore decision-making processes behind vaccination behaviors in older adults.

**Disclosures:**

Gitte Lee Mortensen, n/a, Pfizer: Advisor/Consultant|Pfizer: Grant/Research Support|Pfizer: Honoraria|Sanofi: Advisor/Consultant|Sanofi: Grant/Research Support|Sanofi: Honoraria Oliver Martyn, MPH, Sanofi: Employee

